# Supplementary material for: Modeling glioblastoma heterogeneity as a dynamic network of cell states
Source: Mol Syst Biol. 2021 Sep 16;17(9):e10105. doi: 10.15252/msb.202010105 (PMC8444284; doi:10.15252/msb.202010105)
Supplement: Supplementary file 5 — Source Data for Figure 3 [file MSB-17-e10105-s001.zip › Figure3A_sourcedata/GSEA_3065/hallmarks_state2.GseaPreranked.1623416269049/HALLMARK_EPITHELIAL_MESENCHYMAL_TRANSITION.html]

Details for gene set HALLMARK\_EPITHELIAL\_MESENCHYMAL\_TRANSITION[GSEA]

|  || Dataset | state2 |
| Phenotype | NoPhenotypeAvailable |
| Upregulated in class | na\_neg |
| GeneSet | HALLMARK\_EPITHELIAL\_MESENCHYMAL\_TRANSITION |
| Enrichment Score (ES) | -0.7520746 |
| Normalized Enrichment Score (NES) | -2.9445307 |
| Nominal p-value | 0.0 |
| FDR q-value | 0.0 |
| FWER p-Value | 0.0 |
Table: GSEA Results Summary

  

Fig 1: Enrichment plot: HALLMARK\_EPITHELIAL\_MESENCHYMAL\_TRANSITION      
 Profile of the Running ES Score & Positions of GeneSet Members on the Rank Ordered List

  

| PROBE | GENE SYMBOL | GENE\_TITLE | RANK IN GENE LIST | RANK METRIC SCORE | RUNNING ES | CORE ENRICHMENT || 1 | MCM7 |  |  | 13 | 0.626 | 0.0322 | No |
| 2 | TPM4 |  |  | 848 | 0.133 | -0.0502 | No |
| 3 | GADD45A |  |  | 1791 | 0.073 | -0.1474 | No |
| 4 | JUN |  |  | 1805 | 0.072 | -0.1449 | No |
| 5 | MSX1 |  |  | 2551 | 0.046 | -0.2223 | No |
| 6 | RHOB |  |  | 2690 | 0.042 | -0.2349 | No |
| 7 | ENO2 |  |  | 3006 | 0.034 | -0.2669 | No |
| 8 | GAS1 |  |  | 3144 | 0.031 | -0.2799 | No |
| 9 | TFPI2 |  |  | 3215 | 0.029 | -0.2859 | No |
| 10 | CAPG |  |  | 3281 | 0.028 | -0.2913 | No |
| 11 | FGF2 |  |  | 3688 | 0.021 | -0.3338 | No |
| 12 | CAP2 |  |  | 3862 | 0.018 | -0.3514 | No |
| 13 | WIPF1 |  |  | 3879 | 0.018 | -0.3522 | No |
| 14 | FERMT2 |  |  | 3980 | 0.016 | -0.3620 | No |
| 15 | ELN |  |  | 4163 | 0.013 | -0.3809 | No |
| 16 | COL16A1 |  |  | 4589 | 0.006 | -0.4262 | No |
| 17 | VEGFA |  |  | 4721 | 0.004 | -0.4400 | No |
| 18 | NID2 |  |  | 5511 | -0.009 | -0.5242 | No |
| 19 | NNMT |  |  | 5544 | -0.009 | -0.5271 | No |
| 20 | SNTB1 |  |  | 5960 | -0.016 | -0.5708 | No |
| 21 | ITGB5 |  |  | 6093 | -0.018 | -0.5840 | No |
| 22 | PVR |  |  | 6162 | -0.019 | -0.5903 | No |
| 23 | PDLIM4 |  |  | 6744 | -0.030 | -0.6510 | No |
| 24 | GPX7 |  |  | 6828 | -0.032 | -0.6581 | No |
| 25 | EFEMP2 |  |  | 6895 | -0.034 | -0.6634 | No |
| 26 | PLAUR |  |  | 6906 | -0.034 | -0.6626 | No |
| 27 | LAMA1 |  |  | 7052 | -0.038 | -0.6762 | No |
| 28 | FBLN1 |  |  | 7061 | -0.038 | -0.6750 | No |
| 29 | TIMP3 |  |  | 7309 | -0.045 | -0.6991 | No |
| 30 | TPM1 |  |  | 7414 | -0.048 | -0.7077 | No |
| 31 | COL5A3 |  |  | 7491 | -0.050 | -0.7131 | No |
| 32 | SDC1 |  |  | 7783 | -0.059 | -0.7412 | No |
| 33 | PFN2 |  |  | 7867 | -0.062 | -0.7468 | No |
| 34 | LOXL2 |  |  | 7871 | -0.062 | -0.7438 | No |
| 35 | TNFRSF12A |  |  | 7931 | -0.064 | -0.7467 | No |
| 36 | COLGALT1 |  |  | 7959 | -0.065 | -0.7461 | No |
| 37 | SLC6A8 |  |  | 8016 | -0.068 | -0.7484 | Yes |
| 38 | TPM2 |  |  | 8019 | -0.068 | -0.7450 | Yes |
| 39 | BDNF |  |  | 8073 | -0.070 | -0.7470 | Yes |
| 40 | BMP1 |  |  | 8090 | -0.071 | -0.7449 | Yes |
| 41 | MATN2 |  |  | 8102 | -0.071 | -0.7422 | Yes |
| 42 | SFRP1 |  |  | 8103 | -0.071 | -0.7384 | Yes |
| 43 | FBN1 |  |  | 8107 | -0.072 | -0.7349 | Yes |
| 44 | THY1 |  |  | 8139 | -0.074 | -0.7343 | Yes |
| 45 | ITGA5 |  |  | 8155 | -0.075 | -0.7319 | Yes |
| 46 | SDC4 |  |  | 8172 | -0.076 | -0.7295 | Yes |
| 47 | PLOD3 |  |  | 8187 | -0.077 | -0.7269 | Yes |
| 48 | CXCL12 |  |  | 8251 | -0.080 | -0.7294 | Yes |
| 49 | NOTCH2 |  |  | 8358 | -0.086 | -0.7362 | Yes |
| 50 | FUCA1 |  |  | 8399 | -0.089 | -0.7357 | Yes |
| 51 | PLOD2 |  |  | 8403 | -0.089 | -0.7313 | Yes |
| 52 | PRRX1 |  |  | 8427 | -0.091 | -0.7289 | Yes |
| 53 | TGFB1 |  |  | 8497 | -0.095 | -0.7312 | Yes |
| 54 | MEST |  |  | 8534 | -0.099 | -0.7297 | Yes |
| 55 | PLOD1 |  |  | 8592 | -0.102 | -0.7304 | Yes |
| 56 | COL11A1 |  |  | 8598 | -0.103 | -0.7254 | Yes |
| 57 | COPA |  |  | 8599 | -0.103 | -0.7198 | Yes |
| 58 | TAGLN |  |  | 8662 | -0.108 | -0.7207 | Yes |
| 59 | LGALS1 |  |  | 8680 | -0.110 | -0.7167 | Yes |
| 60 | PCOLCE |  |  | 8685 | -0.110 | -0.7112 | Yes |
| 61 | VIM |  |  | 8691 | -0.110 | -0.7058 | Yes |
| 62 | GJA1 |  |  | 8728 | -0.114 | -0.7036 | Yes |
| 63 | QSOX1 |  |  | 8731 | -0.114 | -0.6977 | Yes |
| 64 | TGFBI |  |  | 8761 | -0.117 | -0.6945 | Yes |
| 65 | P3H1 |  |  | 8767 | -0.118 | -0.6887 | Yes |
| 66 | ID2 |  |  | 8781 | -0.120 | -0.6837 | Yes |
| 67 | NTM |  |  | 8787 | -0.120 | -0.6778 | Yes |
| 68 | THBS1 |  |  | 8789 | -0.120 | -0.6715 | Yes |
| 69 | ADAM12 |  |  | 8799 | -0.122 | -0.6659 | Yes |
| 70 | FLNA |  |  | 8824 | -0.126 | -0.6617 | Yes |
| 71 | MYLK |  |  | 8862 | -0.131 | -0.6587 | Yes |
| 72 | APLP1 |  |  | 8911 | -0.137 | -0.6565 | Yes |
| 73 | CADM1 |  |  | 8914 | -0.137 | -0.6494 | Yes |
| 74 | EMP3 |  |  | 8918 | -0.137 | -0.6423 | Yes |
| 75 | CDH11 |  |  | 8983 | -0.146 | -0.6414 | Yes |
| 76 | PPIB |  |  | 8991 | -0.147 | -0.6342 | Yes |
| 77 | WNT5A |  |  | 9037 | -0.156 | -0.6307 | Yes |
| 78 | SERPINH1 |  |  | 9054 | -0.159 | -0.6239 | Yes |
| 79 | COL4A2 |  |  | 9069 | -0.161 | -0.6168 | Yes |
| 80 | SGCB |  |  | 9101 | -0.166 | -0.6112 | Yes |
| 81 | COL6A2 |  |  | 9116 | -0.170 | -0.6036 | Yes |
| 82 | COL5A1 |  |  | 9148 | -0.177 | -0.5975 | Yes |
| 83 | FSTL1 |  |  | 9158 | -0.179 | -0.5889 | Yes |
| 84 | HTRA1 |  |  | 9178 | -0.184 | -0.5810 | Yes |
| 85 | SERPINE2 |  |  | 9186 | -0.186 | -0.5718 | Yes |
| 86 | NT5E |  |  | 9208 | -0.194 | -0.5637 | Yes |
| 87 | LOXL1 |  |  | 9237 | -0.206 | -0.5557 | Yes |
| 88 | IGFBP2 |  |  | 9240 | -0.208 | -0.5448 | Yes |
| 89 | PTX3 |  |  | 9249 | -0.212 | -0.5343 | Yes |
| 90 | CDH2 |  |  | 9253 | -0.215 | -0.5231 | Yes |
| 91 | CALU |  |  | 9278 | -0.223 | -0.5137 | Yes |
| 92 | SAT1 |  |  | 9279 | -0.224 | -0.5018 | Yes |
| 93 | COL4A1 |  |  | 9284 | -0.226 | -0.4901 | Yes |
| 94 | LAMC1 |  |  | 9286 | -0.226 | -0.4781 | Yes |
| 95 | MMP2 |  |  | 9307 | -0.235 | -0.4676 | Yes |
| 96 | PMEPA1 |  |  | 9313 | -0.241 | -0.4553 | Yes |
| 97 | COL5A2 |  |  | 9316 | -0.244 | -0.4424 | Yes |
| 98 | GPC1 |  |  | 9326 | -0.250 | -0.4300 | Yes |
| 99 | LRP1 |  |  | 9338 | -0.259 | -0.4173 | Yes |
| 100 | ITGAV |  |  | 9346 | -0.269 | -0.4036 | Yes |
| 101 | PMP22 |  |  | 9347 | -0.271 | -0.3891 | Yes |
| 102 | DPYSL3 |  |  | 9354 | -0.276 | -0.3750 | Yes |
| 103 | CD59 |  |  | 9358 | -0.283 | -0.3601 | Yes |
| 104 | FN1 |  |  | 9364 | -0.294 | -0.3449 | Yes |
| 105 | PTHLH |  |  | 9375 | -0.313 | -0.3292 | Yes |
| 106 | DKK1 |  |  | 9377 | -0.313 | -0.3125 | Yes |
| 107 | SPARC |  |  | 9384 | -0.332 | -0.2954 | Yes |
| 108 | TIMP1 |  |  | 9387 | -0.334 | -0.2777 | Yes |
| 109 | ITGB1 |  |  | 9388 | -0.339 | -0.2595 | Yes |
| 110 | CALD1 |  |  | 9391 | -0.348 | -0.2411 | Yes |
| 111 | VCAN |  |  | 9396 | -0.364 | -0.2221 | Yes |
| 112 | TNC |  |  | 9403 | -0.383 | -0.2022 | Yes |
| 113 | GLIPR1 |  |  | 9404 | -0.387 | -0.1815 | Yes |
| 114 | CD44 |  |  | 9407 | -0.397 | -0.1604 | Yes |
| 115 | SPP1 |  |  | 9415 | -0.436 | -0.1378 | Yes |
| 116 | DST |  |  | 9422 | -0.485 | -0.1125 | Yes |
| 117 | IGFBP3 |  |  | 9424 | -0.502 | -0.0858 | Yes |
| 118 | POSTN |  |  | 9437 | -0.750 | -0.0469 | Yes |
| 119 | SCG2 |  |  | 9440 | -0.879 | -0.0000 | Yes |
Table: GSEA details [plain text format]

  

Fig 2: HALLMARK\_EPITHELIAL\_MESENCHYMAL\_TRANSITION: Random ES distribution      
 Gene set null distribution of ES for **HALLMARK\_EPITHELIAL\_MESENCHYMAL\_TRANSITION**
